# Implementation fidelity of a multifactorial in-hospital fall prevention program and its association with unit systems factors: a single center, cross-sectional study

**DOI:** 10.1186/s12913-023-09157-5

**Published:** 2023-02-15

**Authors:** Regula Wyss-Hänecke, Susanne Knüppel Lauener, Constantin Sluka, Mieke Deschodt, Flaka Siqeca, René Schwendimann

**Affiliations:** 1 Institute of Nursing Science, University of Basel, Bernoullistrasse 28, 4056 Basel, Switzerland; 2 MediZentrum Burgergut, Bernstrasse 107, 3613 Steffisburg, Switzerland; 3 Medizinische Direktion, University Hospital of Basel, Hebelstrasse 2, 4031 Basel, Switzerland; 4 Department of Clinical Research, University of Basel and University Hospital of Basel, Spitalstrasse 8/12, 4031 Basel, Switzerland; 5 Department of Public Health and Primary Care, Gerontology and Geriatrics, KU Leuven, Herestraat 49, 3000 Leuven, Belgium; 6 Competence Center of Nursing, University Hospitals Leuven, Herestraat 49, 3000 Leuven, Belgium

**Keywords:** Accidental falls, Preventive health services, Patient safety, Implementation science, Routinely collected health data, Fall prevention program, Implementation outcomes

## Abstract

**Background:**

Falls are a common, costly global public health burden. In hospitals, multifactorial fall prevention programs have proved effective in reducing falls’ incidence; however, translating those programs accurately into daily clinical practice remains challenging. This study’s aim was to identify ward-level system factors associated with implementation fidelity to a multifactorial fall prevention program (StuPA) targeting hospitalized adult patients in an acute care setting.

**Methods:**

This retrospective cross-sectional study used administrative data on 11,827 patients admitted between July and December 2019 to 19 acute care wards at the University Hospital Basel, Switzerland, as well as data on the StuPA implementation evaluation survey conducted in April 2019. Data were analysed using descriptive statistics, Pearson’s coefficients and linear regression modelling for variables of interest.

**Results:**

The patient sample had an average age of 68 years and a median length of stay of 8.4 (IQR: 2.1) days. The mean care dependency score was 35.4 points (ePA-AC scale: from 10 points (totally dependent) to 40 points (totally independent)); the mean number of transfers per patient -(e.g., change of room, admission, discharge) was 2.6 (range: 2.4– 2.8). Overall, 336 patients (2.8%) experienced at least one fall, resulting in a rate of 5.1 falls per 1’000 patient days. The median inter-ward StuPA implementation fidelity was 80.6% (range: 63.9–91.7%). We found the mean number of inpatient transfers during hospitalisation and the mean ward-level patient care dependency to be statistically significant predictors of StuPA implementation fidelity.

**Conclusion:**

Wards with higher care dependency and patient transfer levels showed higher implementation fidelity to the fall prevention program. Therefore, we assume that patients with the highest fall prevention needs received greater exposure to the program. For the StuPA fall prevention program, our results suggest a need for implementation strategies contextually adapted to the specific characteristics of the target wards and patients.

**Supplementary Information:**

The online version contains supplementary material available at 10.1186/s12913-023-09157-5.

## Background

Falls are a global public health burden and the second most common accidental cause of death worldwide [1]. The World Health Organization defines a fall as *“an event which results in a person coming to rest inadvertently on the ground or floor or other lower level”* [1]. Every year, 37.3 million people need medical attention due to injuries resulting from falls; 646,000 die from those injuries [1]. In hospitals, estimates of the global incidence of patient falls range from 5.7 to 18 per 1,000 bed-days [2]; and in Swiss hospitals, where a recent study reported that 29.7% of all acute care patients had a known risk of falling, 3.5% experienced falls [3]. Empirical findings showed that 30–50% of in-hospital falls result in fall-related injuries, such as wounds, contusions or fractures [3, 4].

Multifactorial fall prevention programs have proved effective in reducing fall risks and decreasing fall rates by up to 30% [2, 4, 5]. The most common components of such programs include patient education, bedside risk signs, staff education, multidisciplinary event-review after fall occurrences, stable footwear, alert wristbands, toileting schedules, medication review, environmental modifications, movement alarms, bedrails or low beds and physical exercise. Structured fall risk assessment, sitters to supervise patients continuously, system changes and assistive support have also reportedly been integrated into multifactorial fall prevention programs [2, 4, 6]. In addition, team simulation with interdisciplinary communication about fall prevention and patient engagement appeared to be beneficial, as well as daily multidisciplinary team huddles addressing patients fall risks as well [7, 8]. Still, while these programs’ effectiveness has been demonstrated, little is known about their implementation into daily clinical practice and their long-term sustainability [6, 9].

One of an intervention or care program’s major implementation outcomes is fidelity, i.e., the degree to which, in daily practice, staff perform the related procedures as prescribed by the developers [10–12]. In a systematic review, Hempel et al. [6] showed that higher fidelity to fall prevention programs increased their intervention effects [6]. Research findings often reveal broad gaps between fall prevention programs’ test results and their levels of implementation, adoption and sustainability in clinical practice [9]. Fidelity to fall prevention programs varies widely (from 48 to 90%), with results suggesting relationships to system factors, e.g., clinical specialty, number of patients at risk for falling, and nurse staffing [9, 13].

To map system factors that potentially affect implementation outcomes, the Consolidated Framework for Implementation Research (CFIR) can be used. The CFIR focusses on five key domains of implementation research: the intervention itself, the inner setting, the outer setting, the individuals involved, and the process by which implementation is accomplished [14, 15]. First, researchers need to ensure that the intervention itself fits the targeted problem and the organizational context [14]. Second, the inner setting must be considered, including any relevant organization structural characteristics, e.g., a stable team, desirable manager/employee ratios, decentralized decision making, clear communication, open feedback, and especially compatibility between the intervention, the team’s values, organizational culture and the priority given to the intervention. Further examples include a system for rewarding success, leadership engagement, a learning climate fostered by leadership, an overview of available resources and access to information about the intervention [14]. The outer setting includes aspects of the political, economic and social contexts that can influence the inner setting [14]. Lastly, each involved individual plays a potentially crucial role in the implementation process: each develops a feeling for the intervention that may include emotional involvement. Therefore, individuals may adapt the intervention to their personal needs or values [14]. Successful implementation involves choosing an appropriate intervention, adapting it to the characteristics of the target setting—including any structures with which it will interact—then designing and facilitating implementation processes that fit both the intervention and the target context [14, 16].

To understand how to foster implementation fidelity regarding fall prevention programs to reduce in-hospital falls, we aimed to identify ward-level system factors associated with higher implementation fidelity to our multifactorial fall prevention program targeting hospitalised adults.

## Methods

### Design

This retrospective, cross-sectional study used two data sources: routine administrative data on patients admitted between July and December 2019 in 19 acute care wards at the University Hospital Basel (USB) in Switzerland, and data from the StuPA implementation evaluation survey conducted in April 2019. The study is part of the hospital’s evaluation of their multifactorial fall prevention program “StuPA.”

### Setting and sample

The study was conducted at the USB, a 770-bed tertiary care hospital and one of Switzerland’s five university hospitals. In 2019, 38,000 in-patients were treated on 31 wards, with bed counts ranging from 9 to 48 per ward. Until 2020, the USB had four clinical departments, of which three—the surgical, medical and clinical specialty departments—had bed wards. Of these, eight surgical, seven medical and four specialty clinical wards for adult patients were included in this study (*n* = 19). As StuPA was not developed for high patient turnover contexts, the intensive care units, the intermediate care units, the emergency department and outpatient wards were excluded from our analysis. Five other wards were excluded because they use a different documentation system for fall events, hindering the possibility for data extraction.

### The multifactorial fall prevention program

The USB’s multifactorial, interdisciplinary fall prevention program (StuPA) was implemented in 2013. The program consists of fall risk screening, specific fall prevention interventions and evaluation of fall events for all hospitalised patients, based on the Swiss Patient Safety foundation’s Fall prevention guide [17] (see Appendix A).

StuPA screens for four fall risk factors: age 65 years or older; a history of falls; a nurse-conducted clinical assessment of either gait; or cognition. If all screening questions are answered negatively, patients receive general fall prevention interventions. E.g., ensuring sturdy footwear (including well fitting shoes), adequate mobilisation aids and a safe environment (e.g., light switch; alarm bell and personal belongings reachable; locked bed wheels; etc.). If even one of the screening items indicates a risk for falling, an in-depth fall risk assessment is conducted.

The fall risk assessment is aimed at identifying individual risk factors for falling (e.g., cognitive impairment, vertigo, unsafe gait, impaired vision or intake of medications that increase the risk of falling). Based on the assessment’s results, individual fall prevention interventions are planned and provided. A large set of interventions, e.g., to increase mobility, inform patients about fall risks and preventive measures, review of medication (with physicians), or regular gait training are provided by nurses, who can select and tailor intervention components to individual patients. A re-evaluation of the fall prevention intervention is mandatory when there is a change either in the patient’s clinical status or in the environment (e.g., transfer to another room), or if the patient falls. After a fall, StuPA foresees additional measures to prevent further injury. It also includes a systematic protocol to help document and evaluate the fall. If the patient is still hospitalized the aim is to prevent further falls, while supporting the care team’s practice-based learning.

### Fall prevention program training

During the introduction of the StuPa in 2013, dedicated clinical nurse specialist informed wards nursing staff about the multifactorial fall prevention program via presentations and discussions during grand rounds. In addition, workshops with the ward teams were held to instruct the StuPa algorithm with applying the corresponding items in the patients’ clinical record to assess its fall risk factors, planning and documenting subsequent care interventions incl. post fall measures. Since then, all new hired nurses undergoing the hospitals orientation program, including the StuPa basics within the 2-days training program addressing patient safety issues and other relevant nursing topics. Later on, team huddles at the wards with regard to admitted patients at risk for falling are mentioned and discussed as a appropriate. On wards demand, clinical nurse specialist support the nurses in analysis of falls with injuries with adjusting preventive activities as appropriate. Furthermore, within the hospitals quality and safety assessment strategy, patient safety rounds at the wards with direct observations of the conduct of care activities including those to prevent in-patient falls with feedback to the teams were held [18].

### Data source

The survey data were gathered via a survey conducted in April 2019 to evaluate implementation fidelity to StuPA. The 20-item survey developed by clinical experts, evaluated the fidelity to the StuPA program. The nurse leadership team (nurse manager and clinical nurse specialist) of each ward had to assess the application of all essential fall prevention program components on 4-option Likert-type scales (0.25 = does not apply, 0.5 = tends not to apply, 0.75 = tends to apply, 1 = does apply). The survey items were developed and tailored specifically to evaluate each unit’s fidelity to our local fall StuPA prevention program. The survey’s validity was confirmed by experts in the field.

The survey was completed by the hospital’s chief patient safety officer via a series of structured interviews with each ward’s nursing leadership team.

Between July and December 2019, the relevant administrative data were extracted from routinely collected data in the USB’s patient medical records and the 19 participating wards’ nursing care planning records. In order for data to be included the patient had to be hospitalized during the study period. If a patient had more than one eligible admission during this period, each was included as a separate case. The routine data from the study period were extracted from the hospital’s Clinical Data Warehouse (CDWH), which integrates data from the USB’s primary information systems. The one relevant for this data extraction was the ”Meona” patient medical record system. This contains the “ePA-AC” assessment system used for care plans [19].

### Variables and measurements

#### Outcome variables

StuPA fidelity was assessed based on the survey data. For this study we interpreted higher self-reported ratings of StuPA program components as indicating better implementation fidelity. Three clinical nurse specialists rated and decided together which of the survey’s 20 items would be most relevant for this study (face validity). They chose 12 items, all reflecting essential StuPa components, as relevant to this study’s analysis (see Appendix B).

#### Predictor variables

We extracted data entries to describe eleven key characteristics of each ward and its patients. In addition to *Number of beds*, we used ward admission and discharge dates to calculate the *Number of admitted patients* and the *Length of stay per patient*. We also used four fall-related variables: *Number of falls per patient, Number of patients with a fall risk, Number of patients without a fall risk* and *Number of fall-related injuries.* Four further characteristics were based on case-level data: *Nursing care dependency (NCDY) score at admission (10–40 points (10 points = totally dependent; 40 points = totally independent) *[19]*, Number of cases of patients at risk for delirium, year of birth (i.e., age)* and *number of transfers (admission, discharge, move to another room, absence and readmission after absence)* (see Appendix C).

### Data management

All survey and administrative data were pseudonymized, with only one person from the Department of Clinical Research’s Clinical Data Center having access to identifiable data. Patient data, which included no disclosable information regarding individuals, were analysed at the ward level. All extracted data were stored on a hospital-based, password-secured server.

### Data analysis

Data were analysed using descriptive statistics, linear regression modelling and Pearson’s coefficients. Prior to analysis, data were checked for completeness and plausibility and cleaned. If cases where missing data (NA) occurred, only the variable containing NA was removed, i.e., the entire patient case was not excluded.

We matched the pseudonymized patient cases to their wards. This allowed us to aggregate the data at the ward level. For descriptive statistics regarding the patient data, central tendency was determined by means, medians and ranges. Further frequencies and percentages were analysed per ward for the relevant variables. This allowed us to calculate the *mean number of admitted patients, bed occupancy rates (%)* and *mean length of stay in days* per ward, as well as to present ward-level and mean *percentages of patients with falls, percentages of patients with a fall risk, percentages of falls with fall related injuries* and *number of falls per 1,000 patient days.* In addition to illustrating the wards’ patient characteristics, the *mean nursing care dependency at admission, percentages of patients with a delirium risk, median age of patients and mean number of moves per case per ward* were calculated. Furthermore, an *injury severity score* was calculated by summarizing the severity points (no injuries or NA = 0, minimal injuries = 1, moderate injuries = 2, severe injuries = 3), then expressing the results in relation to the total number of falls per ward.

Each ward’s total StuPA implementation fidelity score was calculated by summing the scores of all surveys, which ranged from 3 to 12 (12 items, each with a maximum value of 1 point = total max. 12 points), then expressing each as a percentage of the total possible sum. For one analysis, we divided the implementation fidelity content into two thematic subgroups: “Clinical Practice fidelity” regarding care staff associated with direct clinical practice, and “Interdisciplinary and Leadership fidelity,” regarding healthcare professionals who are involved as managers and policy makers, but not as clinical practitioners. We then analysed for differences between these subgroups (see Appendix B).

This comparison should allow further insights regarding these StuPA subgroups and their implementation fidelity. For further descriptive analysis we aggregated the wards into “high fidelity wards” (≥ median for total StuPA fidelity) and “low fidelity wards” (< median for total StuPA fidelity).

For our analysis, we used specific ward characteristics—*care dependency at admission* and *mean transfers per case (*both of which we hypothesized would influence StuPA fidelity*)*—as predictor variables and the StuPA implementation fidelity score as our outcome variable. After the statistical requirements were fulfilled, we used Pearson’s product-moment correlation coefficient to test for associations between the variables. Specifically, we assumed that the higher a patients care dependency, the more nursing care interventions apart from fall prevention are needed; therefore, fall prevention receives a lower priority. Equally, regarding patient transfers, as they decrease care consistency, high numbers would lead to lower levels of care. These would include reduced fidelity to a fall prevention intervention.

Finally, to explore whether these two patient-related variables functioned as implementation fidelity predictors, we created and ran a linear regression model. Levels of significance were set at *p* < 0.05.

We performed explorative analysis by plotting other possibly predictive variables in relation to StuPA fidelity. This revealed characteristics that could be interesting for further investigation. We also looked at two single StuPA variables against selected predictive variables as part of an exploratory subgroup analysis.As there are only 19 data points in the survey and the StuPA fidelity score is built from 12 variables, we did not look at all 120 possible combinations with the 10 predictive variables. Instead, we selected two StuPA variables (StuPA_V8 “The fall risk and/or a fall event is taken into account in the patient’s discharge planning (e.g., Info Transfer to Downstream Services)” and StuPA_V9 “Case analyses (VFA) are carried out for all falls with serious injuries.”) and two predictive variables (fall risk and injury severity) as we would expect higher fidelity in these variables on wards with higher fall risk and more severe injuries, respectively. Data analysis was conducted using the R (version 4.0.3) statistical software [20] with the “tidyverse” version 1.3.0 [21] including “plyr” version 1.8.6 data science packages installed [22].

## Results

A total of 11,828 adult patient cases hospitalized in the 19 studied wards were analysed, accounting for 95,914 patient days over the six-month study period. Overall, 336 patients (2.8%) experienced at least one fall, accounting for a total of 491 falls. Therefore, the fall rate was 5.1 per 1,000 patient days; the median rate per unit was 4.5 (range 1.2–10.2) falls per 1,000 patient days. Of the 491 falls, 169 (34.4%) resulted in injuries.

The StuPA fidelity survey dataset was 100% complete (i.e., it had no missing or NA responses). In the “Meona” patient datasets, NA occurred only in the “ePA-AC” care planning system, where it applied to 14% of cases. Regarding the variables used in our analysis, 4.4% of the NCDY responses at admission were NA. For “delirium risk” and “fall risk” at admission, 2.4% of items were answered NA; for fall-related injury severity, the percentage was 10%.

### Sample description

The median patient age was 68 years (range per ward: 53–72 years). The median average length of stay per ward was 8.4 days (IQR: 2.1); and the median bed occupancy rate 89% (IQR: 13%). The median percentage of patient cases per ward with delirium risk at admission was 14.6% (range: 2.9–27%); for cases per ward with fall risk at admission the median was 59% (range: 26–82%). The median of 2% (range: 1.7–5.1%) of patients per ward actually fell during the studied period. For 83% of falls, a fall risk was recognized at admission (range per ward: 0-100%). And a median of 38% (range: 12.5–66.7%) of patients who fell sustained fall-related injuries. For detailed information about the individual wards see also Table 1.

**Table 1:** Ward characteristics (*n* = 19)

Ward	Beds [n] (Cases [n])	StuPA fidelity points [3-12] (%)	Patient transfers [n] (mean per case)	Mean nursing care dependency score at admission [10–40 points] (SD)	Number of falls [n] (Falls per 1000 patient days)	Patient cases with fall risk at admission [n] (%)	Fall Injury severity score [0–3]	Median length of stay [days] (range)	Bed occupancy [%]	Patient cases with delirium risk at admission [n] (%)	Median age [years] (range)
**Surg 1**	37 (553)	10 (77.8)	1‘362 (2.5)	34.4 (2.2)	24 (5.9)	344 (62.2)	0.13	7 (1–49)	59.3	95 (17.2)	62 (17–96)
**Surg 2**	40 (690)	10.75 (86.1)	2‘023 (2.9)	35.4 (5)	37 (6.2)	416 (60.3)	0.65	8 (1–51)	80.9	115 (16.7)	70 (26–97)
**Surg 3**	38 (678)	9.75 (75.0)	1‘635 (2.4)	30.4 (6.7)	15 (2.5)	559 (82.5)	0.33	7 (1-106)	85.6	183 (27)	65 (18–99)
**Surg 4**	34 (669)	10 (77.8)	1‘622 (2.4)	33 (6.4)	25 (4.5)	486 (72.7)	0.2	7 (1–97)	89.8	78 (11.7)	68 (16–101)
**Surg 5**	39 (920)	10.5 (83.3)	2‘436 (2.6)	36 (7.1)	23 (3.5)	336 (36.5)	0.35	6 (1–65)	91.9	99 (10.8)	60 (17–96)
**Surg 6**	36 (1‘064)	9 (66.7)	2‘352 (2.2)	35.9 (6.6)	12 (1.8)	386 (36.3)	0.67	4 (1-127)	99.6	74 (7)	62 (18–102)
**Surg 7**	23 (486)	10 (77.8)	1‘078 (2.2)	34.3 (5.8)	8 (2.2)	298 (61.3)	0.75	6 (1–40)	85.2	58 (11.9)	71 (18–99)
**Surg 8**	20 (445)	10.5 (83.3)	1‘152 (2.6)	33.5 (6.1)	7 (2.1)	319 (71.7)	0.43	6 (1–83)	88.6	97 (21.8)	70 (17–99)
**Med 1**	15 (119)	10.5 (83.3)	385 (2.9)	38.7 (3.4)	8 (3.2)	69 (58)	0.3	27 (2-136)	97.9	15 (12.6)	55 (19–79)
**Med 2**	9 (435)	8.75 (63.9)	1‘207 (2.8)	38.8 (2.9)	2 (1.2)	127 (29.2)	1	3 (1–20)	103.1	17 (3.9)	58 (18–96)
**Med 3**	48 (1‘016)	10.25 (80.6)	2‘619 (2.6)	34.9 (3.4)	77 (8.7)	599 (59)	0.34	7 (1–75)	100.4	225 (22.2)	68 (17–99)
**Med 4**	44 (957)	10.75 (86.1)	2‘875 (3.0)	34.7 (6.9)	48 (6.1)	599 (62.6)	0.65	7 (1–62)	97.5	210 (21.9)	72 (16–97)
**Med 5**	47 (813)	10.25 (80.6)	2‘196 (2.7)	36.3 (6.9)	41 (5.0)	478 (58.8)	0.59	8 (1-180)	94.9	155 (19.1)	68 (16–93)
**Med 6**	44 (827)	11.25 (91.7)	2‘283 (2.8)	35.1 (5.8)	80 (10.2)	517 (62.5)	0.5	8 (1–77)	97.1	177 (21.4)	70 (17–96)
**Med 7**	30 (603)	10.75 (86.1)	1‘627 (2.7)	33 (7.1)	28 (6.1)	454 (75.3)	0.21	7 (1–55)	83.5	155 (25.7)	70 (16–102)
**Spec 1**	25 (495)	10.5 (83.3)	1‘340 (2.7)	36.3 (6.7)	33 (8.1)	240 (48.5)	0.61	7 (1-103)	88.1	72 (14.6)	70 (19–103)
**Spec 2**	24 (649)	10 (77.8)	1‘435 (2.2)	37.8 (6)	6 (2.0)	169 (26)	0.17	3 (1–38)	69.3	21 (3.2)	48 (16–94)
**Spec 3**	25 (826)	9.75 (75.0)	2‘364 (2.9)	38.1 (3.7)	11 (2.7)	239 (28.9)	0.27	3 (1–71)	88.8	36 (4.4)	53 (16–97)
**Spec 4**	9 (348)	9 (66.7)	751 (2.2)	39.2 (8.2)	6 (5.0)	192 (55.2)	0.83	3 (1–17)	72.9	10 (2.9)	69 (18–98)
**Total**	587 (11’827)	-	32‘744	-	491 (5.1)	6‘827 (57.7)	-	NA	88.8	1‘892 (16)	-
**Median**	34 (669)	10.3 (80.6)	1‘627 (2.6)	39 (2.4)	23 (4.5)	344 (59)	0.4	7	88.8	95 (14.5)	68
**Min**	9 (119)	8.8 (63.9)	385 (2.2)	30.4	2 (1.2)	69 (26)	0.1	3	59.3	10 (2.9)	48
**Max**	48 (1‘064)	11.3 (91.7)	2‘875 (3.2)	39.2	80 (10.2)	599 (82.4)	1	27	103.1	225 (27)	72

*n *Number,* SD *Standard deviation,* Surg *Surgical ward,* Med *Medical ward,* Spec *Specialty clinic ward

The median StuPA implementation fidelity of the 19 wards was 80.6% (range: 63.9-91.7%). Wards with a StuPA fidelity below the median of 81% (low fidelity wards) had younger, more independent patients, as well as fewer patients with fall or delirium risk. Additionally, these wards had shorter lengths of stays, lower bed occupancies, fewer patient transfers on the ward and overall lower numbers of patients. Additionally, compared to wards with a StuPA fidelity equalling or exceeding the median (high-fidelity wards), the low-fidelity wards had fewer falls per 1000 patient days and less severe fall related injuries (see also Table 2).

**Table 2:** Characteristics of high and low fidelity wards

	Overall [IQR]	High Fidelity Wards [IQR]	Low Fidelity Wards [IQR]
**N**	19	10	9
**Median Age [years]**	68 [61; 70]	70 [68; 70]	62 [58; 68]
**Mean Length of Stay [days]**	8.4 [7.3; 9.4]	9 [8.5; 10.2]	6.7 [4.9; 7.9]
**Mean Number of Cases treated**	669 [490.5; 826.5]	751.5 [522; 896.8]	649 [486; 678]
**Mean Bed Occupancy [%]**	88.8 [84.4; 97.3]	93.4 [88.2; 97.4]	85.6 [73.0; 89.8]
**Mean Care Dependency [10–40 points] at admission**	35.4 [34.3; 37.1]	35.3 [34.8; 36.2]	35.9 [34.3; 38.1]
**Mean proportion of Patients with Fall risk [%]**	59 [42.5; 62.6]	59.6 [58.2; 62.6]	55.2 [29.2; 62.2]
**Mean Number of Falls per 1000 Patient Days**	4.4 [2.4; 6.1]	6.1 [3.9; 7.7]	2.5 [2.0; 4.4]
**Mean Severity of Fall-Related Injury [0–3 points]**	0.4 [0.3; 0.7]	0.5 [0.4; 0.6]	0.3 [0.2; 0.8]
**Mean Number of Patients with Delirium Risk [%]**	14.6 [8.9; 21.6]	20.2 [15.1; 21.9]	7 [3.9; 11.9]
**Mean Number of Transfers per Patient Case**	2.6 [2.4; 2.8]	2.7 [2.7; 2.9]	2.4 [2.2; 2.5]

High-fidelity wards=those that reported median or higher StuPA fidelity (≥ 81%); Low-fidelity wards=those that reported below-median StuPa fidelity (<81%). The care dependency score [10-40 points] is defined on a continuum: 10 points=totally dependent; 40 points=totally independent patient. For the Injury Severity Score [0-3 points], 0 points=no fall-related injuries; 3 points= every fall resulted in severe fall-related injuries

### Fidelity to the fall prevention program (StuPA)

**Fig. 1:**
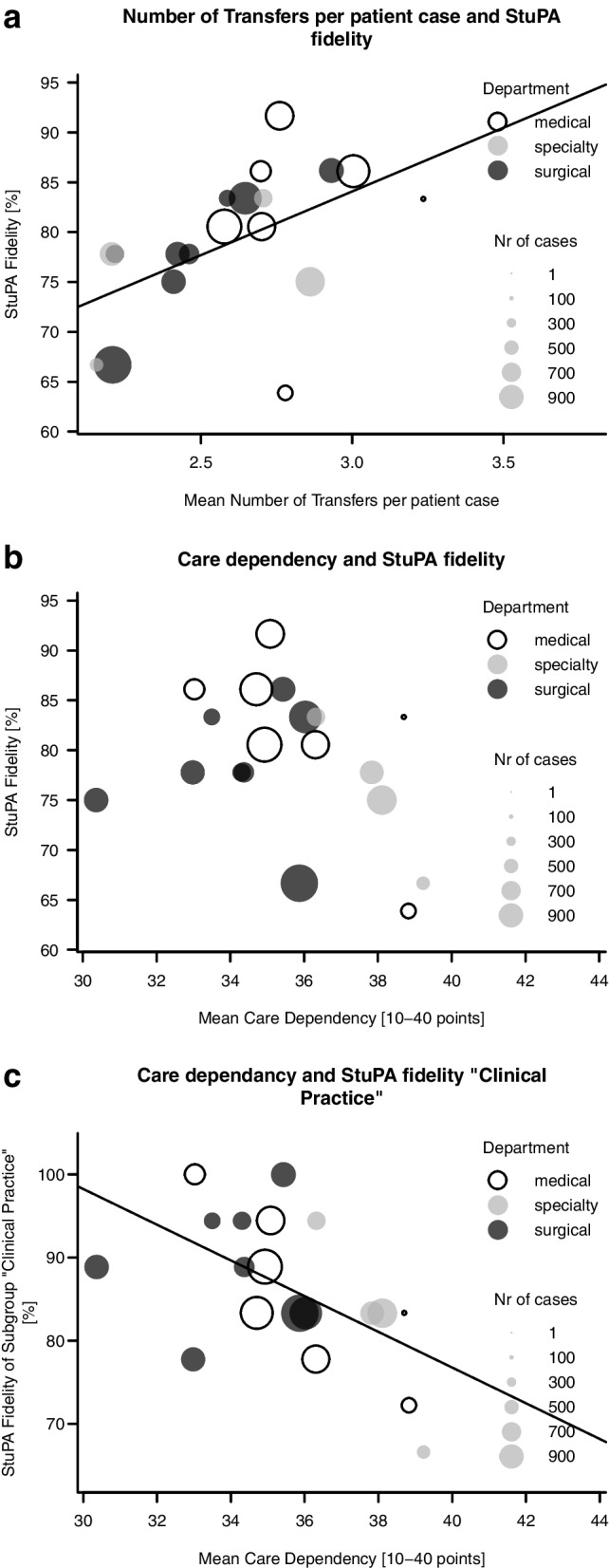
**a** Number of transfers of a patient case and StuPA fidelity. **b** Care dependency and StuPA fidelity. **c** Care dependency and the “Clinical Practice” subgroup’s StuPA fidelity scores. **a-c**The StuPA “Clinical Practice” subgroup includes only items associated with direct clinical practice. Each dot represents one of the 19 wards. Mean Care dependency [10–40 points] is scored on a continuum: 10 points = totally dependent; 40 points = totally independent. These points are shown as means of each ward. Each chart’s grey line represents a simple linear regression of the two variables

Contrary to our hypothesis, Pearson’s product-moment correlation coefficient showed a positive correlation between StuPA fidelity and the mean number of transfers per patient case (*r* = 0.52, *t* = 2.5, df = 17, *p* = 0.24, CI = 95%) (see Fig. 1a). We found no statistically significant correlation (*r*=-0.35, *p* = 0.14, CI = 95%) between overall StuPA fidelity and care dependency (see Fig. 1b). However, regarding the “Clinical Practice” fidelity subgroup, we did find a negative correlation between the StuPA fidelity subgroup “Clinical Practice” and the ward-level patient care dependency scores (*r*=-0,55, *p* = 0.014, CI = 95%) (See Fig. 1c).

In our linear regression model, StuPA fidelity was significantly explained by two ward characteristics: *transfers per patient case* and *care dependency score. Transfers per patient case* appears to have a stronger relationship (see Table 3).

**Table 3:** Linear regression model to predict StuPA fidelity

	Estimate (β)	Standard error	Statistic	*p*-value
**Intercept (β0)**	91.85	22.28	4.1	0.001
**Care dependency score**	-1.44	0.59	-2.45	0,026
**Transfer per patient case**	14.78	4.60	3.2	0.005

The model shows that for each additional point of the *care dependency score* (i.e., 1 point less dependent, because it is an inverse score), StuPA fidelity diminishes by 1.4%. An even stronger effect is seen with *transfers per patient case*. If a ward has 1 extra transfer per case, the fidelity increases by 15%. The equation of the linear regression model is:

$$91.85\;+\;(-1.44)\;{}^{\mathit\ast}\textit{Care dependency score}\:+\:14.78{}^{\mathit\ast}\textit{transfers per case}\:=\:\textit{StuPA fidelity}\;\%$$

### Explorative analysis

**Fig. 2:**
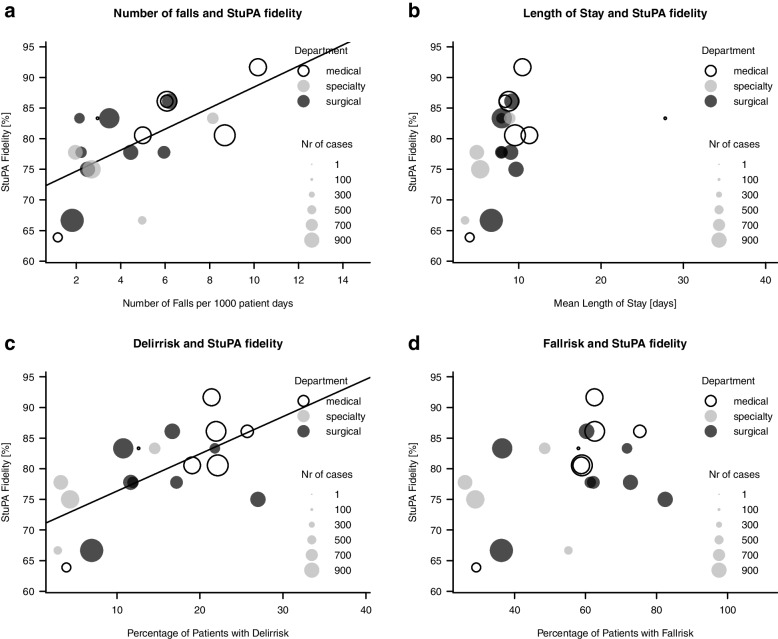
**a-d** Explorative plots of StuPA fidelity and ward characteristics. Each dot represents one of the 19 test wards. For the Injury Severity Score [0–3 points], 0 points = no fall-related injuries occurred: 3 points = every fall resulted in severe fall-related injuries

Here we will present plots of ward characteristics, which yielded interesting results in our explorative analysis. (See Fig. 2).

Regarding exploratory intent, the Pearson’s product-moment correlation coefficient suggests a correlation between StuPA fidelity and the number of falls per 1,000 patient days (*r* = 0.61, *p* = 0.006). Also, the percentage of patients with a delirium risk was positively associated with the StuPA fidelity score (*r* = 0.65, *p* = 0.003).

StuPA fidelity scores tend to be associated neither with wards’ percentages of patients for whom fall risk is indicated (*r* = 0.43, *p* = 0.07), nor with the mean length of patient stay (*r* = 0.42, *p* = 0.07).

Fall-related injury severity showed no association either with the StuPA fidelity score (*r*=-0.38, *p* = 0.1) or with patient age (*r* = 0.36, *p* = 0.1). And neither the wards’ bed occupancy levels nor their numbers of treated cases correlate with StuPA fidelity scores (respectively, *r* = 0.05, *p* = 0.8; *r* = 0.12, *p* = 0.6).

### Exploratory subgroup analysis

**Fig. 3:**
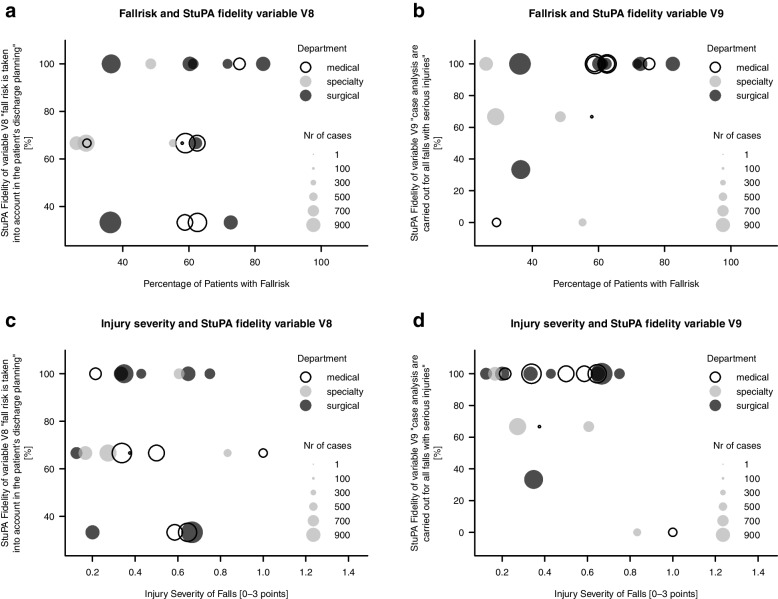
**a**-**d** Explorative plots of StuPA variables V8 and V9 and ward characteristics fall risk and injury severity. Each dot represents one of the 19 test wards. For the Injury Severity Score [0–3 points], 0 points = no fall-related injuries occurred: 3 points = every fall resulted in severe fall-related injuries

The variable StuPA_V8 (*“The fallrisk and /or fall event is taken into account in the patient’s discharge planning”)* shows no correlation with fall risk and injury severity, respectively, while the results for StuPA_V9 *(“case analysis are carried out for all falls with serious injuries”)* are strongly influenced by the two wards with “not true” answers and thus no conclusions should be drawn (Fig. 3).

**Fig. 4:**
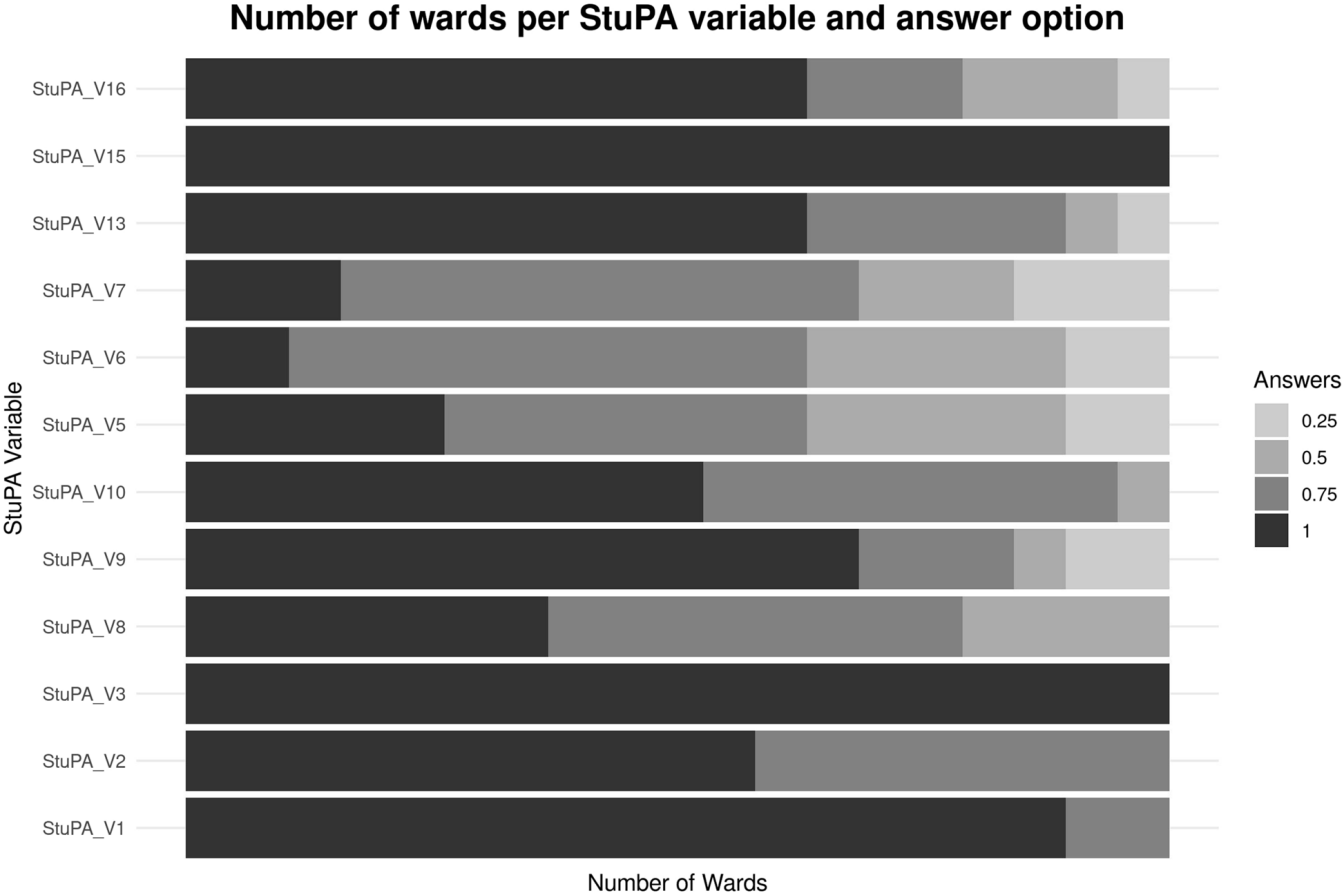
Distribution of answers for individual StuPA variables

Finally, we present an overview on the individual StuPA variables for all wards here (Fig. 4). Variables representing the StuPA subgroup “Interdisciplinarity and Leadership” tended to have lower fidelity scores than those representing the subgroup “Clinical Practice”.

## Discussion

With this retrospective cross-sectional study in 19 university hospital acute care wards, we identified associations between ward-level system factors and implementation fidelity regarding a multifactorial fall prevention program. Across all study wards, we found high implementation fidelity scores regarding the StuPA fall prevention program. The range was between 63.9% and 91.7%, which is above the rates reported in current international and Swiss literature [9, 13]. This means even our study’s “low fidelity wards” have relatively high implementation fidelity; therefore, StuPA should be viewed as successfully implemented overall at the USB.

We observed statistically significant associations between each of two ward characteristics—*transfers per patient case* and *care dependency level*—and StuPA implementation fidelity. Exploratory analysis suggested additional associations between implementation fidelity and both *number of falls per 1,000 patient days* and *percentage of patients with delirium risk*. Furthermore, we were able to map the characteristics of wards with higher and lower StuPA implementation fidelity.

We found a positive correlation between patient care dependency and fidelity to the fall prevention program in the *clinical practice* subgroup, i.e., the higher a ward’s level of patient dependency (the more care they needed), the higher the level of fidelity to the fall prevention program. Here we assume that patients with the highest need for fall prevention interventions receive the most attention regarding such interventions. This principle is addressed in the literature: care dependency in activities of daily living is commonly treated as a fall risk indicator [23]. The StuPA fall prevention program could also be tailored to the specific needs of patients treated on high implementation fidelity wards, which supports high fall prevention program fidelity. On low implementation fidelity wards, adaptions such as providing younger, less care-dependent patients specific information about fall prevention, might be considered.

These findings could also be explained by the Health Belief Model [24] For example, if there is a higher perceived susceptibility and higher benefit for using the intervention, it would make sense that the fidelity is higher. This can also explain why those wards with higher fall rates had higher fidelity.

Our findings also showed a positive correlation between patient transfers and implementation fidelity, i.e., the more patient transfers a ward had, the higher its implementation fidelity. To our knowledge, the association between transfers of patients in a hospital and fall prevention implementation fidelity has not previously been studied. However, studies using dual-task testing, e.g., as part of fall risk assessment, have shown that patient performance was poorer in busy clinical environments compared to in calm environments [25].

As a systemic fall risk factor, a very busy environment has been linked to reductions both in patients’ cognitive processing speed and in their ability to adjust their gait while walking [25]. However, while high patient transfer rates both indicate and increase busyness, care staff in the studied wards did not respond to increased numbers of admissions and discharges by cutting back on their implementation efforts. On the contrary, possibly because they understood the link between high in/out traffic and additional fall risk, they consistently increased their implementation fidelity as traffic increased.

Furthermore, patient transfers within and between hospital wards plausibly indicate fluctuating care needs of the transferred patients. For more complex patients—including those at risk for delirium—this commonly results in high care dependency. The literature includes various reports of associations between patient room transfers or intra-hospital transitions and risks for delirium or adverse events such as falls [26–28]. This is congruent with our findings that, on wards with higher fall rates, higher care dependency and more patient transfers, nurses StuPA responses showed greater fidelity to fall prevention interventions. Moreover, the StuPA data analysis indicates that, if a patient is transferred to another room or ward, their fall risk should be reassessed. If this indicates that such transfers increase identification of fall risks, this knowledge would allow staff to tailor or adjust fall prevention interventions, as they did with fall prevention implementation fidelity in several of the studied wards.

In the explorative analysis, the apparent association between fall prevention implementation fidelity and delirium risk is especially notable: as depicted in Fig. 2c, the more patients at risk for delirium were treated on a specific ward, the higher that ward’s fall prevention implementation fidelity was. This phenomenon was also observed in a recent study [29] involving patients who had both delirium symptoms and significantly higher fall risk. Those whose fall risk was gauged as high received significantly longer periods of delirium-specific care than those with lower fall risks [29]. These findings indicate a need for closer investigation and alignment of fall and delirium prevention programs.

Interestingly, wards with higher fall rates showed higher fall prevention fidelity than those with lower rates. We interpret this relationship as an expression of higher fall prevention awareness on wards with more falls. I.e., it is not surprising that fall prevention has the highest implementation fidelity on wards with the highest need for fall prevention, possibly because their patients are commonly more fall-prone, i.e., are particularly care-dependent. Therefore, such associations do not cast doubt on the StuPA program’s effectiveness. On this matter, based on recent evidence [2, 4, 5], our descriptive findings show that overall, our study wards had a relatively low fall rate per 1,000 patient days [2, 30]. Also, this study’s fall prevention implementation fidelity scores showed narrower ranges than those reported in another study (respectively 63.9–91.7% vs. 48–90%) [9]. This also shows that even our lowest-fidelity wards were still conducting almost 64% of the intervention.

As noted, we observed higher fall prevention implementation fidelity in wards where fall risks were more prevalent; however, none of the observed associations were statistically significant. Therefore, as the fall risk assessment is an integral part of the fall prevention program, we expect that the program fidelity variable is somehow confounded. One likely explanation is that as program fidelity rises, more patients are screened, leading to higher apparent fall risk prevalence. Nevertheless, the risk assessment’s low percentages of NA responses suggest that its results were reliable.

Our descriptive analysis revealed that most of the studied wards have considerably higher percentages of patients with a fall risk (study ward mean: 59%; Swiss mean: 29.7%). Further, 17% of this study’s falls were by patients whose admission assessment did not indicate any particular risk of falling. I.e., the StuPA fall risk assessment tool lacked the sensitivity to detect their fall risk. In view of targeted and specific interventions, current literature discusses the development of a predictive fall risk assessment tool that will allow more sensitive risk assessment [31]. A more sensitive risk assessment—one including not only a dichotomised (Yes/No) fall risk assessment, but assigning the risk into categories such as no, low- or high-risk [32]—would allow more effective stratification of patient fall risks [32].

Our explorative analysis showed no statistically significant association between patient length of stay and fall prevention implementation fidelity. However, the trend suggests a link between longer mean hospitalization on a ward and higher fall prevention program fidelity. Previous studies did not consider increased lengths of stays when fall prevention programs were conducted [33]. Therefore, we assume that high implementation fidelity does not increase patient stays; instead, longer average stays reflect greater admission values of patients’ complexity and dependency [34]. Both of these characteristics correlate with higher fall risk.

Our findings do not suggest an association between the severity of injurious falls and fall prevention implementation fidelity. The percentage of falls resulting in injuries was comparable both to the Swiss average [3] and to rates reported in international literature [35]. Although we observed 10% missing data regarding fall severity, StuPA was clearly effective at reducing falls; therefore, it also reduced the number, aggregated severity and both human and financial burdens associated with related injuries.

Finally, our results did not show any association between patient age and fall prevention implementation fidelity. According to the current literature, although older patients tend to experience more falls, age alone is not a sufficient fall risk predictor [36]. Stronger associations have been shown between frailty and falls [36]. Therefore, further investigation of associations between patient frailty and fall prevention implementation fidelity is recommended.

The exploratory subgroup analysis for two individual StuPA variables was inconclusive, because the number of four answer options limits the analysis of single StuPA variables.

### Methodological considerations

This study provides an explorative overview of different hospital ward characteristics that might be associated with the implementation fidelity of the StuPA multifactorial fall prevention program. As StuPA was developed using current literature, the results may also be applicable for other multifactorial fall prevention programs within acute care settings. Using data on the total patient population and these wards’ program fidelity over a six-month data collection period, we achieved an informative mapping of the participating wards’ characteristics in relation to the fall prevention program.

Although the fall prevention program fidelity rating points were collected via a structured interview with each ward’s, nursing leadership team, a recall- and/or social desirability bias cannot be ruled out. To improve the value of the fidelity scores, we recommend using individual patient-level data to assess StuPA fidelity, e.g., to record how many interventions each patient received, whether screening was performed, etc. By producing a more precise aggregated total of each ward’s program fidelity points, this would allow more active identification of—and correction for—biased reporting. However, such adaptions are outside the scope of the current study.

The use of routine data can bring an additional risk of sampling bias. However, we used a large patient sample with relatively few NA responses in the dataset; and overall, only one analysed variable had more than 5%: fall-related injury severity yielded 10% NAs.

Furthermore, this study’s cross-sectional study design does not allow any inferences of causality.

Finally, one must also consider that implementation fidelity could have been linked to characteristics not examined in this study. Possibilities include individual staff characteristics (e.g., educational level, professional experience of nurses or nurse/patient ratios), or leadership components (leadership engagement; available resources and access to information about the intervention; a stable team [9, 13] or interdisciplinary cooperation [30]). The existence and details of such possibilities could be explored in further research [37].

## Conclusion

StuPA intervention implementation fidelity was high across all studied wards. Those with more care-dependent patients and more patient transfers showed higher implementation fidelity. Our results suggest that wards with more complex patients—and thus a higher overall fall risk—show greater sensitivity to fall prevention, translating both to higher implementation fidelity regarding fall prevention programs and to greater numbers of patients considered at risk. In such wards, one plausible assumption is that all patients who show any signs of fall risks receive the intervention.

Implications for clinical practice include the possibility for in-depth evaluation of the fall prevention program components, which would inform adaptations to the specific needs of high- and low-fidelity wards. Also, regarding low-fidelity wards, as falls are relatively infrequent in such wards, other clinical challenges are higher-priority targets. For such wards, a pared-down version of the StuPA fall prevention program would likely be sufficient. There is also a possibility that other fall prevention resources, e.g., specific patient information and tactics, e.g., stakeholder involvement, could be more useful on the low fidelity wards. Particularly on low implementation fidelity wards, fall prevention interventions should be tailored to the patient population’s specific needs.

Further research will be needed to investigate fall risk assessment practices, whether (and if so, how) patient transfers are related to fall prevention implementation fidelity and other possible fall-influencing factors. In our study a relatively high percentage of patients’ admission assessments indicate risk for falling; others with no indication of fall risk also fall. This prompts question regarding both the used fall risk assessment’s sensitivity and the interventions chosen in response to a positive indication. As the study data comes from 2019 it would be interesting for further research whether the fall prevention program’s fidelity changed during Covid-19 pandemic.

## Supplementary Information

**Additional file 1: Appendix A.** Multifactorial fall preventionprogram (StuPa) Components with Nursing Interventions/Activities. **Appendix B.** Outcome variables. **Appendix C.** Variables WardCharacteristics.

## Data Availability

The datasets used and analysed for the current study are available from the corresponding author upon reasonable request. The fall related routine data from patient medical records and the participating ward’s nursing care planning records can be independently requested from the University Hospital of Basel Clinical Data Warehouse (CDWH).

## Abbreviations

CDWH: Clinical data warehouse
CFIR: Consolidated framework for implementation research
CI: Confidence interval
df: Degrees of freedom
ePA-AC: Care planning system
IQR: Interquartile range
Meona: Primary patient record system used at USB
n: Number
NA: Missing data
r: Correlation coefficient
SD: Standard deviation
NCDy: Nursing care dependency
StuPA: Multifactorial fall prevention program at university hospital Basel
t: t-value
USB: University Hospital Basel
