# Supplementary material for: Implementation fidelity of a multifactorial in-hospital fall prevention program and its association with unit systems factors: a single center, cross-sectional study
Source: BMC Health Serv Res. 2023 Feb 15;23:158. doi: 10.1186/s12913-023-09157-5 (PMC9930071; doi:10.1186/s12913-023-09157-5)
Supplement: Supplementary file 1 — Additional file 1: Appendix A. Multifactorial fall preventionprogram (StuPa) Components with Nursing Interventions/Activities. Appendix B. Outcome variables. Appendix C. Variables WardCharacteristics. [file 12913_2023_9157_MOESM1_ESM.docx]

# ADDITIONAL FILES

**Appendix A – Multifactorial fall prevention program (StuPa) Components with Nursing Interventions/Activities**

**Screening of all patients at admission for risk of falls**

- History of falls (in the last 6 months)?

- Unsafe gait (e.g., unsteady, weak gait)?

- Cognition (e.g., confused, forgetful)

- Patient >65 years?

**Assessment of patients considered at risk for falling**

- Altered cognition/mood (Mental status)?

- Impaired mobility (gait, balance, neurological function)?

- Circumstances and consequences of earlier falls?

- Altered elimination and use of toilet?

- Examine patients for acute or chronic medical condition(s)

- Impaired vision?

- Dizziness/Syncope, circulatory problems?

- Medications potentially increasing the risks of falls (e.g. Sedatives, Hypnotics)?

**Interventions for all patients to provide safety in the hospital**

- Orient patients to surroundings/"set up" of bed room

- Place call bell and personal belongings within reach

- Keep bed in low position

- Provide nightlight at bedside

- Ensure sturdy footwear or Anti-Slip socks

- Lock wheels on wheelchairs, beds, bed tables and night commodes

- Provide walking aids (devices) are fitted and used appropriately

**Additional interventions in patients considered at risk for falling**

- Inform patients (family) about fall risk factors and interventions

- Ensure regular patient surveillance as appropriate

- Use half-length bed side rails instead of full length side rails

- Provide sensor matt at the bedside as appropriate

- Consider delirium management according to the hospital’s standard of care

- Toilet patient regularly

- Assist unsteady patient with ambulating and walking

- Refer patients for gait/balance training, exercise program with occupational therapist

- Review and modify medication with physician

- Inform other care settings after discharge about patient’s fall risk

**Post fall measures**

- Calming the patient and check for injuries

- Provide first aid (incl. X-ray), and monitoring clinical deterioration as appropriate

- Evaluate circumstances of the fall

- Reassessment of patient’s risk factors for falls

- Provide fall report (protocol)

- Continue/adapt interventions to modify patients fall risk

**Appendix B – Outcome variables**

| variable name | variable lable | answer options |
| --- | --- | --- |
| StuPA_V1  Subgroup „Clinical Practice“ | Beginning at admission, a fall risk assessment (ePA-AC) is performed daily for all patients during their stay in hospital. | «not true» (=0.25),  «rather not true» (=0.5),  «rather true» (=0.75),  «true» (=1) |
| StuPA_V2  Subgroup „Clinical Practice“ | For patients at risk of falling, measures are taken to prevent falls (care planning). | «not true» (=0.25),  «rather not true» (=0.5),  «rather true» (=0.75),  «true» (=1) |
| StuPA_V3  Subgroup „Clinical Practice“ | All in-patient falls are systematically recorded (fall protocol). | «not true» (=0.25),  «rather not true» (=0.5),  «rather true» (=0.75),  «true» (=1) |
| StuPA_V8  Subgroup „Clinical Practice“ | The fall risk and/or a fall event is taken into account in the patient's discharge planning (e.g., Info Transfer to Downstream Services) | «not true» (=0.25),  «rather not true» (=0.5),  «rather true» (=0.75),  «true» (=1) |
| StuPA_V9  „Clinical Practice“ Subgroup | Case analyses (VFA) are carried out for all falls with serious injuries. | «not true» (=0.25),  «rather not true» (=0.5),  «rather true» (=0.75),  «true» (=1) |
| StuPA_V10  „Clinical Practice“ Subgroup | Patients at risk of falling are encaged/involved in their prevention of falls. | «not true» (=0.25),  «rather not true» (=0.5),  «rather true» (=0.75),  «true» (=1) |
| StuPA_V5  „Interdisciplinarity and Leadership”  Subgroup | The physiotherapists are actively involved in the prevention of falls. | «not true» (=0.25),  «rather not true» (=0.5),  «rather true» (=0.75),  «true» (=1) |
| StuPA_V6  „Interdisciplinarity and Leadership”  Subgroup | Physicians are actively involved in the prevention of falls. | «not true» (=0.25),  «rather not true» (=0.5),  «rather true» (=0.75),  «true» (=1) |
| StuPA_V7  „Interdisciplinarity and Leadership”  Subgroup | The delirium resource nurses are actively involved in the prevention of falls | «not true» (=0.25),  «rather not true» (=0.5),  «rather true» (=0.75),  «true» (=1) |
| StuPA_V13  „Interdisciplinarity and Leadership”  Subgroup | Sitting guards are instructed in fall prevention measures. | «not true» (=0.25),  «rather not true» (=0.5),  «rather true» (=0.75),  «true» (=1) |
| StuPA_V15  „Interdisciplinarity and Leadership”  Subgroup | The ward managers/nursing supervisors receive regular reports on fall occurrence. | «not true» (=0.25),  «rather not true» (=0.5),  «rather true» (=0.75),  «true» (=1) |
| StuPA_V16  „Interdisciplinarity and Leadership”  Subgroup | The StuPA program management supports the department in the StuPA  implementation. | «not true» (=0.25),  «rather not true» (=0.5),  «rather true» (=0.75),  «true» (=1) |
| StuPA_ward | Sum of the four answer options’ points for StuPA V1, V2, V3, V8,  V9, V10, V5, V6, V7, V13, V15, V16 in relation to the total possible points on each ward | 3–12 points and percentages |
| StuPA_ward „Clinical Practice“ | Sum of the four answer options’ points for StuPA V1, V2, V3, V8,  V9, V10 in relation to the total possible points for these variables. | Percentages |
| StuPA_ward „Interdisciplinarity and Leadership” | Sum of the four answer options’ points for StuPA V5, V6, V7, V13, V15, V16 in relation to the total possible points for these variables | Percentages |

Percentages of scores are calculated by dividing the effective score (actual score - minimum possible score) by the maximum effective score (maximum possible score - minimum possible score) and multiplying the result by 100.

**Appendix C– Variables Ward Characteristics**

*Overview of patient related data assessed. This data was aggregated to each ward which allowed us to calculate ward characteristics*

| variable | label | answer options |
| --- | --- | --- |
| Age | age (calculated: 2019-year of birth) | Integer numbers |
| Length of stay | length of stay in days (calculated by admission date – discharge date for each ward) | Integer numbers |
| Bed occupancy | total hospitalisation days on the ward (calculated by adding up the number of patients staying on the ward on each day of the study period) divided by the number of beds on the ward, multiplied by the duration of study period in days (184 days) | Percentages |
| Care dependency score | nursing care dependency scale (ePA-AC(19)) (10 [totally dependent]–40 [totally independent] points). | Integer numbers |
| Transfers of patient | number of transfers of a patient (admission, discharge, movement to another room on the same or different ward) | Integer numbers |
| Fall rate | number of falls divided by total number of patient days in the study period for each ward | Float numbers |
| Fall risk | fall risk (in ePA-AC)  (and calculated percentages of patients on the ward who have a fall risk in %) | yes = 1 no = 0  (%) |
| Delirium risk | delirium risk (in ePA-AC)  (and calculated percentages of patients on the ward with a delirium risk in %) | yes = 1 no = 0  (%) |
| Patients with a fall | min. 1 fall during hospitalization in patient record  (and calculated percentages of patients with a fall out of all patients on the ward in %) | yes = 1 no = 0  (%) |
| Number of falls | number of falls by all patients divided by the number of patient days | Integer numbers |
| Fall related injuries | fall-related injuries during hospitalization in patient record | no injuries or NA = 0 minimal injuries = 1 moderate injuries = 2 severe injuries = 3 |
| Injury severity score | sum of injury points (as described above) on the ward, divided by total number of falls | 0-3 |
